# A CAF-Based Two-Cell Hybrid Co-Culture Model to Test Drug Resistance in Endometrial Cancers

**DOI:** 10.3390/biomedicines11051326

**Published:** 2023-04-29

**Authors:** Raed Sulaiman, Pradip De, Jennifer C. Aske, Xiaoqian Lin, Adam Dale, Kris Gaster, Luis Rojas Espaillat, David Starks, Nandini Dey

**Affiliations:** 1 Department of Pathology, Avera Research Institute, Sioux Falls, SD 57105, USA; 2 Translational Oncology Laboratory, Avera Research Institute, Sioux Falls, SD 57105, USAjennifer.aske@avera.org (J.C.A.); adam.dale@avera.org (A.D.); 3 Department of Internal Medicine, University of South Dakota SSOM, USD, Sioux Falls, SD 57105, USA; 4 Viecure, Greenwood Village, CO 80111, USA; 5 Assistant VP Outpatient Cancer Clinics, Avera Cancer Institute, Sioux Falls, SD 57105, USA; 6 Department of Gynecologic Oncology, Avera Research Institute, Sioux Falls, SD 57105, USA

**Keywords:** ex vivo resistance platform, tumor–TME cellular model, CAF-mediated resistance, laboratory friendly

## Abstract

The management of advanced or recurrent endometrial cancers presents a challenge due to the development of resistance to treatments. The knowledge regarding the role of the tumor microenvironment (TME) in determining the disease’s progression and treatment outcome has evolved in recent years. As a TME component, cancer-associated fibroblasts (CAFs) are essential in developing drug-induced resistance in various solid tumors, including endometrial cancers. Hence, an unmet need exists to test the role of endometrial CAF in overcoming the roadblock of resistance in endometrial cancers. We present a novel tumor–TME two-cell ex vivo model to test CAF’s role in resisting the anti-tumor drug, paclitaxel. Endometrial CAFs, both NCAFs (tumor-adjacent normal-tissue-derived CAFs) and TCAFs (tumor-tissue-derived CAFs) were validated by their expression markers. Both TCAFs and NCAFs expressed positive markers of CAF, including SMA, FAP, and S100A4, in varying degrees depending on the patients, while they consistently lacked the negative marker of CAF, EpCAM, as tested via flow cytometry and ICC. CAFs expressed TE-7 and immune marker, PD-L1, via ICC. CAFs better resisted the growth inhibitory effect of paclitaxel on endometrial tumor cells in 2D and 3D formats compared to the resistance of the tumoricidal effect of paclitaxel in the absence of CAFs. TCAF resisted the growth inhibitory effect of paclitaxel on endometrial AN3CA and RL-95-2 cells in an HyCC 3D format. Since NCAF similarly resisted the growth inhibitor action of paclitaxel, we tested NCAF and TCAF from the same patient to demonstrate the protective action of NCAF and TCAF in resisting the tumoricidal effect of paclitaxel in AN3CA in both 2D and 3D matrigel formats. Using this hybrid co-culture CAF and tumor cells, we established a patient-specific, laboratory-friendly, cost-effective, and time-sensitive model system to test drug resistance. The model will help test the role of CAFs in developing drug resistance and contribute to understanding tumor cell-CAF dialogue in gynecological cancers and beyond.

## 1. Introduction

In advanced staged endometrial cancers, the critical challenge, such as in most solid tumors, remains the management of the disease due to the development of resistance to therapy and relapse despite surgery, hormone therapy, and chemotherapy. Resistance to ongoing therapy is attributed to intrinsic refractoriness and resistant tumor cells’ emergence during treatment within a short time frame [1]. More than 25% of patients with endometrial cancers are diagnosed at a stage > 1, with an invasive primary tumor, and subsequently, the conditions progresses as a metastatic disease [2]. The most commonly practiced treatment strategies for endometrial cancers involve surgery, followed by a combination of chemotherapy (paclitaxel, carboplatin/cisplatin, and doxorubicin/liposomal doxorubicin), radiation therapy alone or combined with hormonal therapy (American Cancer Society guidelines). According to the NCCN guidelines, the FDA-approved targeted therapies include multi-TKI, lenvatinib, and anti-angiogenic bevacizumab, with the recent inclusion of immunotherapy, pembrolizumab with lenvatinib, or dostarlimab for high TMB, MSI-high dMMR tumors.

The tumor microenvironment (TME) has emerged as an essential critical factor in tumor progression in solid tumors, including gynecological tumors [3]. Once inducted by tumor cells, CAFs engage with both tumor cells and all other components of the TME towards the progression of the disease and the worst outcome. As the most abundant stromal cells within TME, CAFs have a specific role in developing drug resistance in various solid tumors [4,5].

A tumor–CAF liaison exploits the host’s stroma in favor of tumor progression in which CAF–tumor cell cross-talk in concert with the rest of the components of the TME as abettors of resistance to treatment, which is the bête noire of therapy [6]. We briefly overviewed the origin, activation, markers, and overall functions of CAF, with a particular reference to how different functions of CAF in an established tumor are functionally connected to the development of resistance to cancer therapy in solid tumors acting as the roadblock to therapy [7]. The current literature established the undeniable role of CAF in conversation with tumor cells and the rest of the components of TME in mediating the development of treatment resistance and a poorer outcome caused by the disease [8]. Recently, we have studied the role of CAFs in the development of resistance to the anti-angiogenic drug in ovarian cancers [9].

CAF-initiated corrupted signaling exists as part of a bi-directional conversation of CAFs with tumor cells [10]. We reviewed one example of mechanistic interactions between tumor cells and CAFs via the Wnt pathway, which acts as *Un Colpevole Comune* (a common culprit) in these cancers [11]. Given this fact, there is an urgent need to gain knowledge about CAF–tumor cell conversation, which will empower us to improve the outcome of the disease through CAF-inclusive therapy. To engage in CAF–tumor dialogue, we need to know about CAF–tumor conversation in a patient-specific manner. Hence, there is an unmet need to design a model for testing the role of endometrial CAFs in overcoming roadblocks of resistance to therapy to manage the disease. Here, we present a novel tumor–TME two-cell patient-specific ex vivo model to test CAFs’ role in resisting anti-tumor drugs in a patient-specific manner. Using this hybrid model of co-culture (HyCC) of endometrial CAFs and tumors, we established a patient-specific, laboratory-friendly, cost-effective, and time-sensitive testing system, *N-of-1*, to test the role of CAFs in mediating the development of resistance to paclitaxel.

## 2. Materials and Methods

### 2.1. Patient Consent and Tissue Collection

The institutional and/or licensing committee approved all experimental protocols. Informed consent(s) (IRB approved: Protocol Number Study: 2017.053-100399_ExVivo001) was obtained from 53 patients. Resected, unfixed tumor tissue(s) and tumor-adjacent normal tissue(s) were collected from the pathology department. Tissues were collected during surgery in designated collection media as per the guidelines and relevant regulations and provided by the pathologist, depending upon the availability of the tissue on a case-by-case basis. Samples were collected in DMEM/F-12 + Glutamax 500 mL (base) supplemented with HyClone Penicillin-Streptomycin 100 × 100 mL (1%).

### 2.2. Cell Lines and Reagents

Human uterine fibroblasts (HUF; Primary Uterine Fibroblasts, Cat #PCS-460-010) and endometrial cells (RL-95-2 and AN3CA) were procured from ATCC and were cultured according to standard cell culture procedures. Antibodies for immunocytochemistry (ICC) were bought from Cell Marque, NOVUS, Abcam, Agilent-Dako, and Cell Signaling. All cells were used within 7–8 passages and tested negative for mycoplasma. The antibodies for WB were procured from Cell Signaling, USA.

### 2.3. Expression of CAF Markers via Flow Cytometry

**Figure 1:**
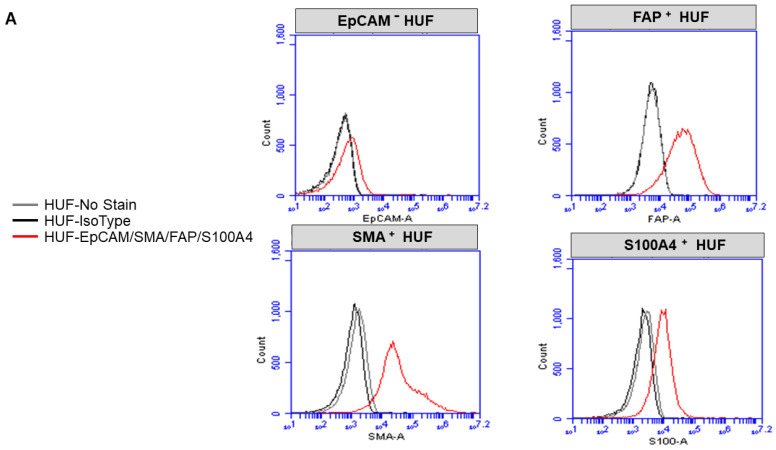

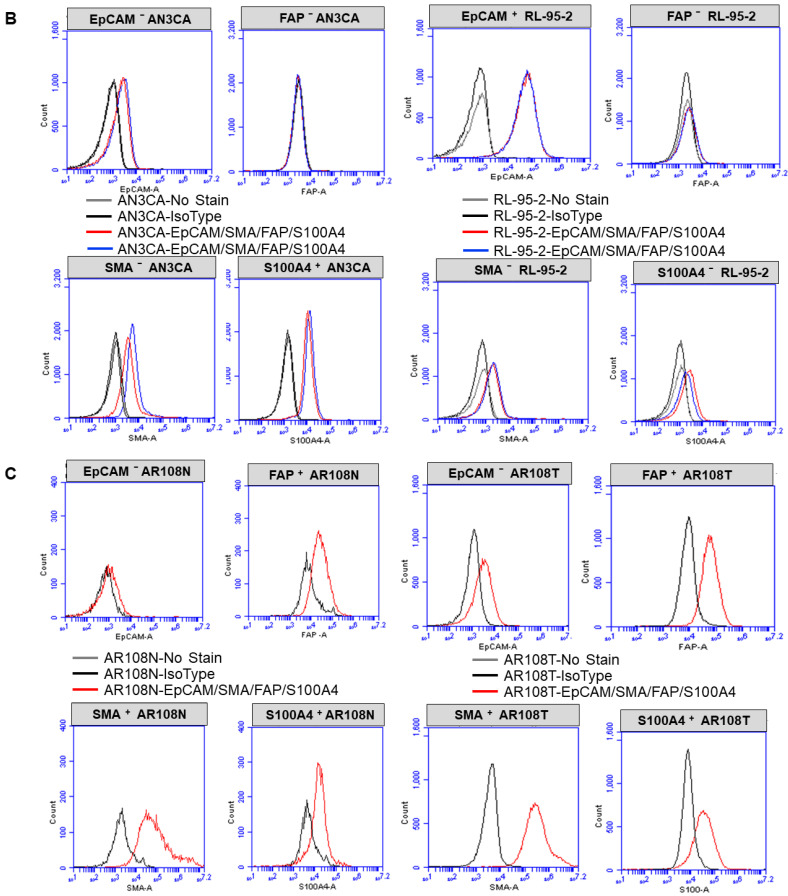

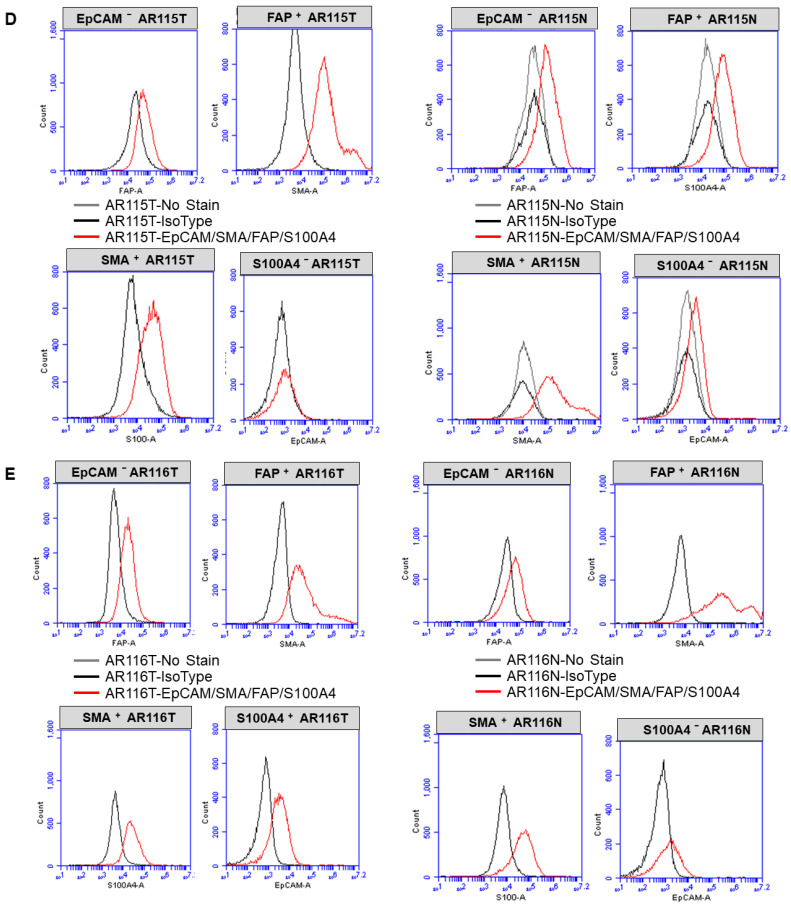
Characterization of enzyme digestion independently cultured CAF via flow cytometry: Flow cytometric (**A**–**E**) evaluation of marker proteins for CAF was conducted. Expression of positive and negative controls of CAF are tested in internal controls of HUF (**A**) and two endometrial cell lines, AN3CA and RL-95-2 (**B**). Both NCAFs and TCAF were characterized by flow cytometric expression of EpCAM, FAP, SMA, and S100A4 in three representative patients with endometrial cancers (**C**–**E**). The grey line represents no stain, the black line represents iso-type controls, and the red line represents the protein expression of choice.

Cells were trypsin released and rinsed in FACS buffer (1% FBS in phenol red-free RPMI). The cell number was adjusted to 1 × 10^6^ cells per sample and resuspended in FACS buffer along with corresponding cell surface antibodies (FAP R&D systems, PD-L1 Miltenyi) and incubated at 4 °C for 20 min. Cells were rinsed twice with FACS buffer, then fixed for 30 min, rinsed, and resuspended in permeabilization buffer (Fix/Perm kit Miltenyi), and the corresponding intracellular antibodies (SAM and S100A4) were added for 30 min. Cells were again rinsed and resuspended in FACS buffer and analyzed on an Accuri C6 cytometer. FCS Express (DeNovo software) was used for analysis (Figure 1). Flow cytometry was performed using SMA-FITC, FAP-PE, S100A4-PerCP, and EpCAM-APC. Cells were stained for 15 min with cell surface antibodies or corresponding isotype control antibodies (Miltenyi). TCAFs and NCAFs from all patients with established CAFs at every passage of the primary culture were tested for the expression of markers to confirm specificity.

### 2.4. Characterization of CAF via ICC

**Figure 2:**
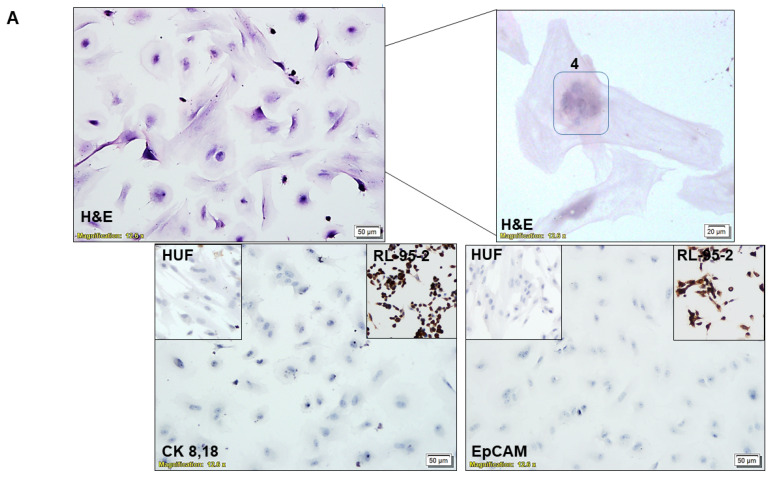

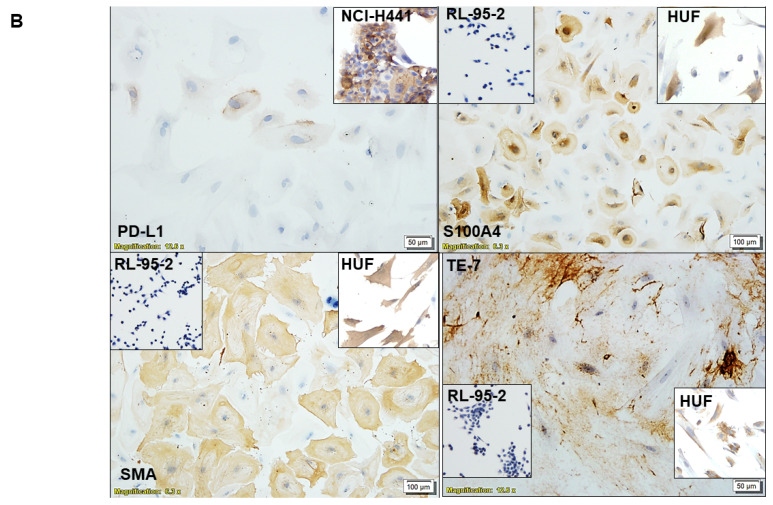
Characterization of enzyme digestion independently cultured CAF via ICC: Multi-nuclear CAF (H&E; number in the inset refers to the number of nuclei) were negative for the expression of epithelial markers, EpCAM, and CK 8,18 (**A**). The number “4” in sub-figure A denoted the number of nuclei in the CAF. The expression of the proteins in RL-95-2 was used as the positive control, and expression in HUF was used as the negative control (insets). Expression of positive CAF markers, SMA, S100A4, TE-7, and immune marker PD-L1 are presented (**B**). The expression of the proteins in RL-95-2 was used as negative controls, and expressions in HUF and NCI-H441 were used as positive controls (insets).

We characterized the CAFs by testing the subcellular expression of EpCAM, CK 8,18, SMAalpha, S100A4, TE-7, and PD-L1 (Figure 2). ICC was performed with relevant positive controls and negative controls and was evaluated by a pathologist. We used (1) endometrial tumor cells, RL-95-2 and AN3CA, as the positive control for epithelial cells; (2) HUF cells as the positive control for fibroblast cells; (3) endothelial cells, HUVEC as the negative control for EpCAM, CK 8,18, SMAalpha, S100A4, and TE-7 and as the positive control for CD31; (4) NCI-H441 cells as the positive control for PD-L1; (5) MCF7 cells were used as the negative control for PD-L1. The list of antibodies used is presented elsewhere [12].

### 2.5. Establishment of Patient-Specific Ex Vivo Tumor–TME Two-Cell Model of Hybrid Co-Culture (HyCC)

**Figure 3:**
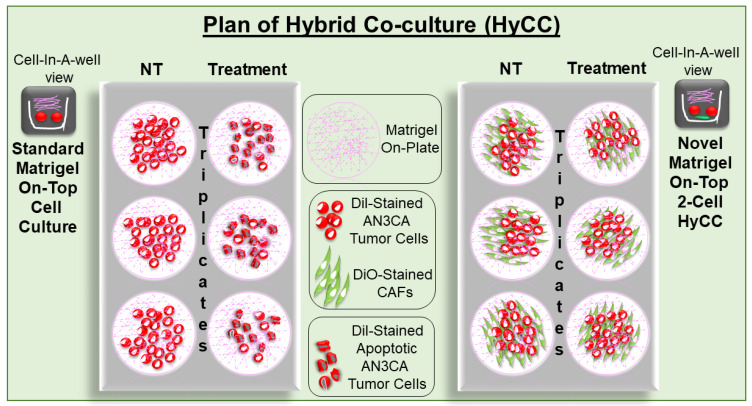
Schematic plan of the Hybrid Co-Culture (HyCC): The novel matrigel 2-cell HyCC of CAFs stained with DiO- and DiI-stained AN3CA cells was presented as compared to the standard matrigel cell culture for 3D clonogenic growth of cancer cells. The model was used to test the effect of the cytotoxic drug paclitaxel on tumor cells.

We designed a novel matrigel-based two-cell HyCC model comprising patient-derived primary pre-characterized CAFs and tumor cells. Figure 3 presents a diagrammatic representation of the detailed plan of the HyCC in our study. We used DiO and DiI, two family members of lipophilic fluorescent stains used to label cell membranes and other hydrophobic structures, as long-term tracer dyes. DiO stain is a green, fluorescent, lipophilic carbocyanine dye that is widely used as a lipophilic tracer. DiI is a lipophilic orange-red fluorescent dye that diffusely stains the entire cell, and it is spectrally similar to tetramethylrhodamine. The HyCC consisted of a preparatory phase and an experiment phase. The preparatory phase of the HyCC was composed of (1) the validation of positive and negative CAF markers via flow cytometry, ICC, and qRT-PCR (depending on the number of CAFs available in the same passage from which the HyCC set up); (2) the staining of verified TCAFs and NCAFs and endometrial tumor cells, AN3CA and RL-95-2 by DiO and DiI stain, respectively; (3) the testing of fluorescence signals in cells via flowcytometry and immuno-fluorescence; (4) the comparison of 2D and 3D growth patterns of unstained and stained cells; (5) the comparative testing of the effect of paclitaxel on 2D and 3D growth patterns between unstained and stained cells. The experiment phase was composed of (1) the overnight plating of DiO-CAFs on plates to obtain an 80–95% confluent monolayer of CAF; (2) the plating of DiI-stained tumor cells on DiO-CAFs with and without paclitaxel in 2D and 3D formats. Media were changed every 48 h. Double-fluorescence signals from the live cells in cultures were recorded, along with bright field photo-micrographs at different time points using dry objectives in Olympus BX43 Microscope using cellSens 1.18 LIFE SCIENCE IMAGING SOFTWARE (OLYMPUS CORPORATION). Semi-quantification was performed based on the fluorescence intensities of DiI tumor cells on DiO-TCAFs/NCAFs from 5–6 random microscopic fields of independent experiments (performed in quadruplicates). Statistical significance was determined by calculating Student’s *t*-test at *p* < 0.05.

### 2.6. Testing Resistance of CAFs to Paclitaxel Using Hybrid Co-Culture of Tumor–TME Two-Cell Model

First, we tested the growth of AN3CA and RL-95-2 in HyCC on TCAF in 2D and 3D formats. Then, we tested the effect of CAF on resisting the tumoricidal effect of paclitaxel in (1) AN3CA in HyCC on TCAF in the 3D format, (2) RL-95-2 in HyCC on TCAF in the 3D format, (3) AN3CA in HyCC on NCAF in the 3D format, (4) RL-95-2 in HyCC on NCAF in the 3D format and (5) AN3CA in HyCC on both NCAF and TCAF from the same patient’s samples in both 2D and 3D formats. Media were changed every 48 h. Double-fluorescence signals from the live cells in cultures were recorded, along with bright field photo-micrographs at different time points using dry objectives in Olympus BX43 Microscope using cellSens 1.18 LIFE SCIENCE IMAGING SOFTWARE (OLYMPUS CORPORATION). Semi-quantification was performed based on the fluorescence intensities of DiI tumor cells on DiO-TCAFs/NCAFs from 5–6 random microscopic fields of independent experiments (performed in quadruplicates). Significance was determined by calculating Student’s *t*-test at *p* < 0.05.

## 3. Results

### 3.1. Ex Vivo Primary Culture- and Marker-Based Verification of CAF

The institutional and/or licensing committee approved all experimental protocols. Informed consent(s) (IRB approved Protocol Number Study: 2017.053-100399_ExVivo001) was obtained from 53 consecutive patients with endometrial tumors at any stage/grade of the disease undergoing surgery with or without a pre-treatment/history of any previous carcinoma. Primary cultures of CAFs were created from the resected samples of tumors and tumor-adjacent normal samples from patients with endometrial cancers, as per the guidelines and relevant regulations provided by the pathologist who performed grossing.

Validation of TCAF and NCAF in the ex vivo primary culture of the patient’s samples was performed. Characterization of the cultured TCAF (from the resected samples of tumor samples) and NCAF (from the resected tumor-adjacent normal samples) via flow cytometry (Figure 1A–E) and ICC (Figure 2A,B) was performed. Both TCAFs and NCAFs expressed positive markers of CAF, including SMA, FAP, and S100A4, to a varying degree, depending on the patients, but they were consistently negative for negative markers of CAF, EpCAM (a positive marker of epithelial tumor cells). The expression of S100A4 was qualitative in a patient-specific manner, with it being absent in some patients, as represented in Figure 1. The expression of positive and negative controls of CAF were tested in internal controls, HUF (A), and two endometrial cell lines, AN3CA and RL-95-2 (B).

CAFs have the morphology characteristic of fibroblasts with a single nucleus, although we sometimes observed multinucleated CAFs, as represented in Figure 2A’s upper panel. CAFs were negative for the ICC expression of epithelial markers, EpCAM and CK 8,18 (Figure 2A’s lower panel). The ICC expression of EpCAM and CK 8,18 in RL-95-2 was used as the positive control, and the ICC expression in HUF was used as the negative control (insets). The CAFs ICC expressed positive fibroblast markers, SMA, S100A4, TE-7, and immune marker, PD-L1 (Figure 2B). The ICC expression of SMA, S100A4, TE-7, and PD-L1 proteins in RL-95-2 was used as the negative controls, and expressions in HUF (for SMA, S100A4, and TE-7) and NCI-H441 (for PD-L1) were used as the positive controls (insets).

### 3.2. Patient-Specific Ex Vivo Tumor–TME Two-Cell Model of Hybrid Co-Culture (HyCC)

**Figure 4:**
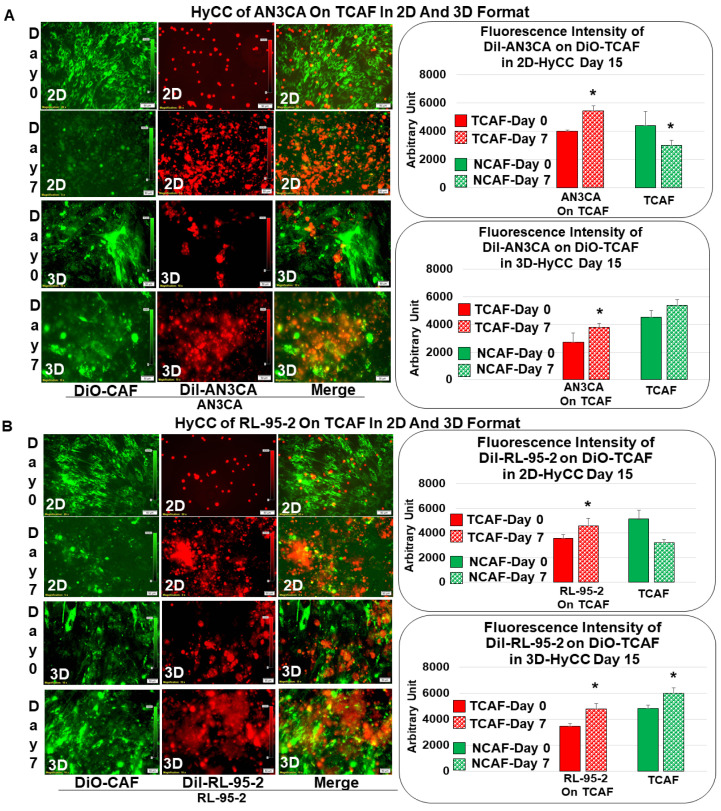
Growth of endometrial cells AN3CA and RL-95-2 for TCAF on TCAF in HyCC in 2D and 3D formats: Dil-stained AN3CA (**A**) and RL-95-2 (**B**) were plated on DiO-stained TCAF, and their 2D and 3D matrigel growth was recorded. The media were changed every 48 h. Photomicrographs were taken on day 0 (within 24 h for the 3D format as pictures are difficult to focus at zero hours in 3D) and on day 7. The fluorescence intensity of tumor cells on TCAF was semi-quantified from 5 random microscopic fields of independent experiments (performed in quadruplicates). Solid bars represent the fluorescence intensities of cells at day zero, and filled (sphere) bars represent the fluorescence intensities of cells at day 7. * Significance was determines by calculating Student’s *t*-test at *p* < 0.05.

We first tested the growth pattern of endometrial tumor cells in 2D and 3D formats of HyCC on TCAFs, as diagrammatically presented in Figure 3. The photomicrographs in Figure 4A,B show that the growth of AN3CA and RL-95-2 was significantly more extensive on day 7 compared to day 0, which was semi-quantified by the fluorescence intensity of the DiI-stained area in both 2D and 3D formats. The DiO-CAFs’ fluorescence intensities, on the other hand, in the 3D format, were either increased or not significantly changed after 7 days. In contrast, the fluorescence intensity of DiO-CAF was found to be reduced in the 2D format. Figure 3 compares the standard model of the matrigel 3D clonogenic growth of cancer cells with the HyCC presented in the “Cell-In-A-Well View”, which was designed to test the functional role of CAFs on the effect of the cytotoxic drug paclitaxel on tumor cells.

### 3.3. Testing the Effect of CAFs in Resisting Paclitaxel Using Ex Vivo Platform-Based Co-Culture of Tumor–TME Two-Cell Model

To test the effect of CAFs on the growth inhibitory effect of paclitaxel on endometrial cells, first, we tested the HyCC growth pattern of these cells in 2D and 3D formats (Figure 4A,B). Dil-stained AN3CA (A) and RL-95-2 (B) were plated on DiO-stained TCAF, and their 2D and 3D matrigel growth was recorded. Media were changed every 48 h. Photomicrographs were taken on day 0 (within 24 h for the 3D format as pictures are difficult to focus at zero hours in 3D) and on day 7. The fluorescence intensity of tumor cells on TCAF was semi-quantified from five random microscopic fields of independent experiments (performed in quadruplicates). Both AN3CA and RL-95-2 growth was significantly increased after 7 days of culturing. However, the clonogenic 3D growth of AN3CA is characteristically different in terms of morphology from that of RL-95-2 cells.

**Figure 5:**
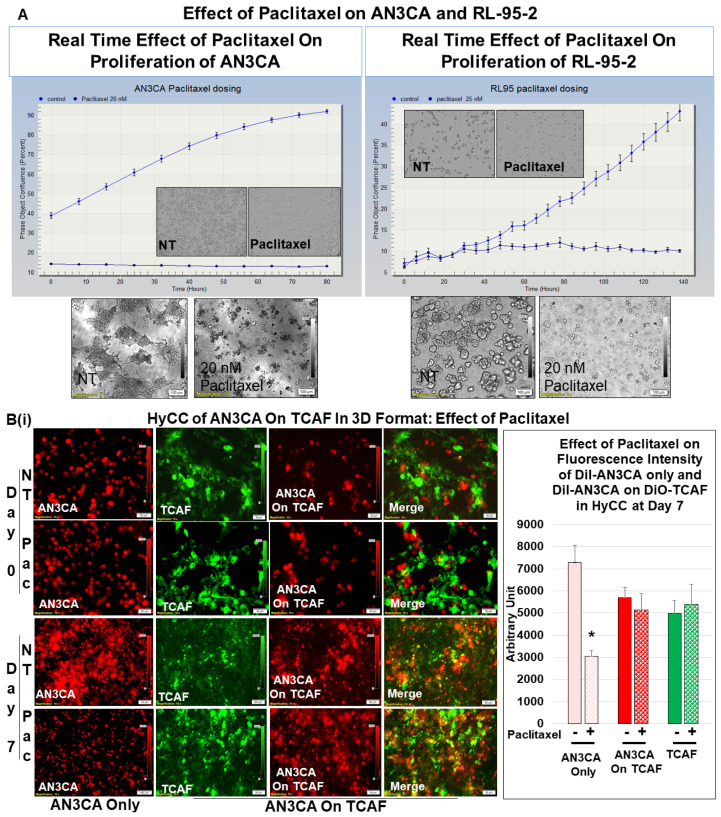

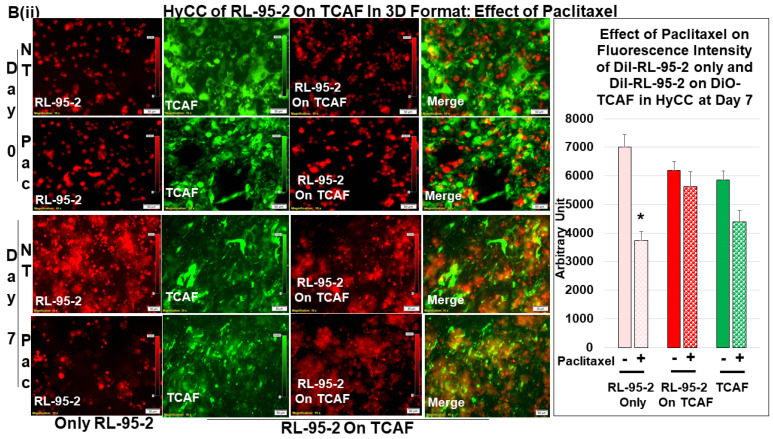
Effects of paclitaxel on the growth of endometrial tumor cells cultured with or without TCAF: Growth inhibitory effect of paclitaxel in real-time 2D growth and 3D on-matrigel growth of AN3CA and RL-95-2 is presented (**A**). Effect of paclitaxel on the same cells co-cultured on the TCAFs (**B**). TCAF resisted the growth inhibitory effect of paclitaxel on AN3CA (**Bi**) and RL-95-2 (**Bii**) in an HyCC 3D format. The media were changed every 48 h. The fluorescence intensity of tumor cells on TCAF was semi-quantified from 5 random microscopic fields of independent experiments (performed in quadruplicates). Solid bars represent the fluorescence intensities of cells at day zero, and filled (sphere) bars represent the fluorescence intensities of cells at day 7. * Significance was found by calculating Student’s *t*-test at *p* < 0.05.

**Figure 6:**
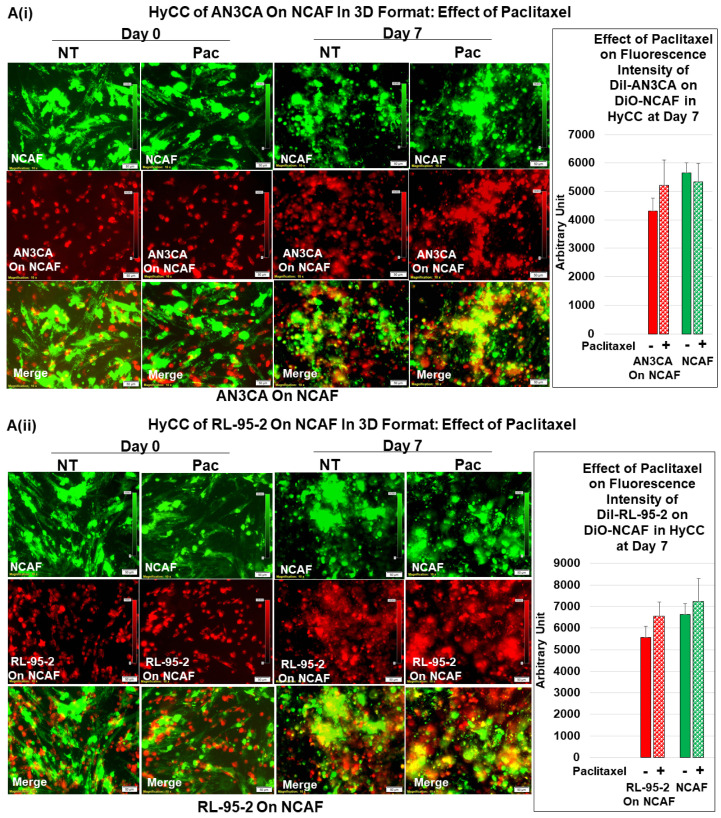
Effects of paclitaxel on the growth of endometrial tumor cells cultured on NCAF: Effect of paclitaxel on the endometrial cells co-cultured on the NCAFs (**A**). NCAFs resisted the growth inhibitory effect of paclitaxel on AN3CA (**Ai**) and RL-95-2 (**Aii**) in an HyCC 3D format. The media was changed every 48 h. The fluorescence intensity of tumor cells on TCAF was semi-quantified from 5 random microscopic fields of independent experiments (performed in quadruplicates). Solid bars represent the fluorescence intensities of cells at day zero, and filled (sphere) bars represent the fluorescence intensities of cells at day 7.

**Figure 7:**
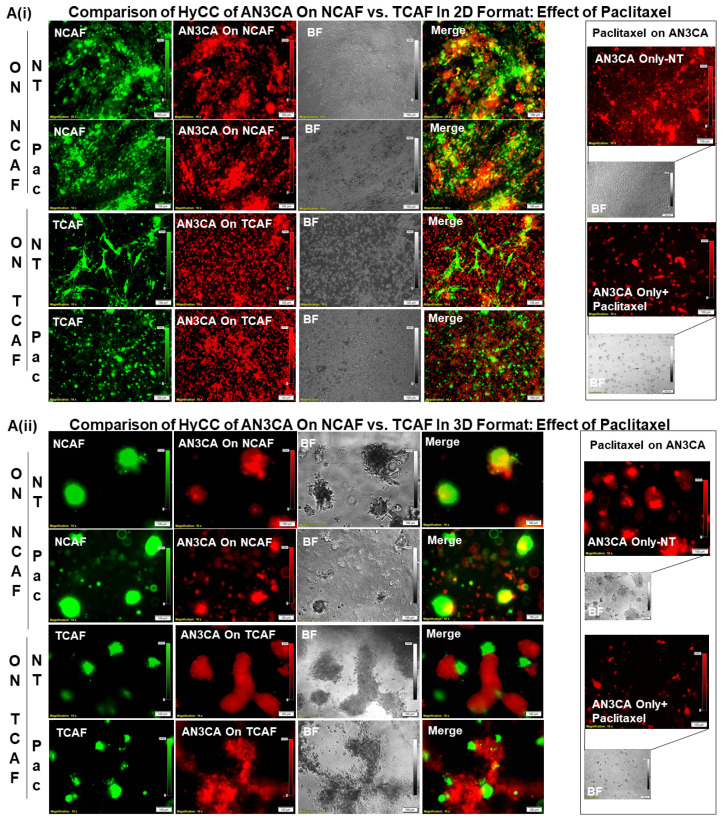
Effects of paclitaxel on the growth of endometrial tumor cells cultured on NCAF and TCAF of the same patient: Protective Action of NCAF-P4 and TCAF-P5 of the same patient in resisting the tumoricidal effect of paclitaxel in AN3CA in 2D (**Ai**) and 3D matrigel (**Aii**) formats are presented. Insets (**Ai**,**Aii**) show the growth inhibitory effect of paclitaxel in DiI-stained regular AN3CA cells, which were not plated on CAFs (without HyCC). The media were changed every 48 h. The fluorescence intensity of tumor cells on TCAF was semi-quantified from 5 random microscopic fields of independent experiments (performed in quadruplicates).

Once we established regular growth patterns of the cells via HyCC, we tested the effect of CAFs on the growth-inhibitory action of paclitaxel. The treatment with paclitaxel decreased the growth of both AN3CA and RL-95-2 in real time (2D), as well as in the 3D format, as shown in Figure 5A. This growth inhibitory property of paclitaxel was used as the baseline to measure the role of CAF in resisting the effect of paclitaxel on tumor cells. Having ascertained the growth pattern of DiI tumor cells of DiO-CAFs in HyCC and tested the growth inhibitory effect of paclitaxel on tumor cells, we sought to test the capacity of CAFs to resist the growth inhibitory effect of paclitaxel on tumor cells. Figure 5(Bi,Bii) shows that TCAF resisted the growth inhibitory effect of paclitaxel on both tumor cells on day 7. The treatment of cells with paclitaxel in the absence of TCAF (AN3CA-only and RL-95-2-only columns of Bi and Bii, respectively) demonstrated the profound loss of growth, as shown in the photomicrographs and bar diagrams in the inset. Once we demonstrated the role of TCAFs in resisting the growth-inhibitory effect of paclitaxel, we wanted to test whether NCAFs from another patient would exhibit a similar effect on TCAFs. Figure 6(Ai, Aii) shows that NCAFs’ capability to resist the growth-inhibitory effect of paclitaxel was comparable to that of TCAFs. Interestingly, we observed no change in the DiO-CAFs, both TCAFs and NCAFs, before and after paclitaxel in the 3D format and the 2D format. We observed that both TCAFs and NCAFs from different patients similarly resisted the growth-inhibitory effect of paclitaxel on endometrial tumor cells. Considering the differences in the pathological characteristics of the tumors from which TCAFs and NCAFs were derived, it is possible that the similar resisting effects (to paclitaxel) observed in both patients were inherent to the characteristics of the TME in those two patients. Hence, from the experiments, it was unclear whether NCAFs and TCAFs behaved similarly due to the underlying pathological differences in the TMEs of the two patients. Therefore, we then tested TCAFs and NCAFs derived from the same patient to demonstrate that both TCAFs and NCAFs resisted the growth-inhibitory effect of paclitaxel in AN3CA cells in both 2D and 3D formats (Figure 7(Ai,Aii)). The insets show the growth inhibitory effect of paclitaxel alone on the tumor cells in the absence of CAFs. We also observed that the DiO-CAFs’ fluorescence intensities in the 3D format were either increased parallel to the growth of tumor cells or did not significantly change after 7 days of HyCC. In contrast, the DiO-CAFs; fluorescence intensities were found to be reduced in the 2D format on every occasion (Figure 4 and Figure 5).

## 4. Discussion

Cancer progression has long been understood to be fundamentally determined and characterized by dynamic changes in signaling between the stroma and embedded tumor cells [13]. CAFs, which are often found in primary and metastatic solid tumors, including endometrial cancers, are highly versatile, plastic, and resilient cells that are essentially involved in cancer progression and treatment failure. As the most involved contributing members of TME by volume, CAFs have an undeniable role in the progression of advanced disease [4,5,14,15] and in the development of treatment resistance in several solid tumors [16,17,18,19]. In the past several years, a number of preclinical and translational studies revealed several functions of CAFs in mediating critical tumor phenotypes to cause a poor outcome for patients with the disease [20]. The functions mentioned above of CAFs are just beginning to reveal an array of direct/indirect interactions with tumor cells and the rest of the components of TME, tumor-associated immune cells, and tumor-associated endothelial cells [21,22,23].

From the point of view of the entirety of the tumor, the role of CAF has been mathematically determined. Benjamin Wölfl et al. mathematically framed the tumor–TME interaction as a model of an evolutionary game [24] to conceptualize and analyze biological interactions where the tumor cells’ fitness is not only influenced by their own genomic traits but also by the traits of cells within the TME, including CAFs. Their model implied that the progression of cancer is an interactive evolutionary competition between these different cell types, which is an interaction that can be explained through Lotka–Volterra competition equations and their extension term, the term “Deadlock game” and the term “Leader game”, in the context of the presence or absence of drugs and/or cancer-associated fibroblasts. Building on the concept, Kaznatcheev et al. [25] demonstrated that cancer-associated fibroblasts qualitatively switch the type of game from “Leader to Deadlock” being played by the in vitro population using their non-small-cell lung cancer model. In their system, they viewed an untreated tumor as being similar to DMSO + CAF, and thus, played the Leader game. A treatment with Alectinib (Alectinib + CAF) or eliminating CAFs (through a stromal-directed therapy) switched the game into a “Deadlock game.” Our data conceptually support their model. Our study experimentally explains their extension terms of “Deadlock game” and “Leader game“. Our model provides an experimental testing platform of the “Deadlock game” mode with a chemotherapy drug in culture, from which it switches to the ‘Leader game’ mode in the presence of CAF (paclitaxel-treated cells plated on CAFs in HyCC). Their report validates our ex vivo two-cell HyCC-based model of drug resistance, which can be used to test clinically viable drug combinations and outcome data in the future.

In lieu of a deterministic role of CAF influencing the disease outcome [4,26], several studies provided evidence that supports CAF-inclusive therapy for the clinical management of the disease [27]. Indeed, 37 CAF-directed/inclusive clinical trials have been currently instituted and are ongoing at the NIH (https://clinicaltrials.gov/ct2/home accessed on 12 December 2022). The majority of these clinical trials are instituted in advanced/metastatic solid tumors (*ClinicalTrials.gov Identifier: NCT05547321*), including pancreatic ductal adenocarcinoma (PDAC), hepatocellular carcinomas, and malignant tumors of the breast, colon, prostate, lung, and ovary, explaining the interest of the scientific community in the clinical relevance of CAF in these organ types, especially in PDAC (*ClinicalTrials.gov Identifier: NCT05262855*). Although CAF-directed clinical trials are just beginning to reveal the clinical relevance of CAFs, the role of CAFs in endometrial cancers, among other gynecological cancers, is surprisingly limited. We have recently reported the first study to prove the clinical relevance of CAF in endometrial cancers [12]. As more of these studies in the future will strengthen our knowledge of CAF signaling in endometrial cancers, we will need to develop a personalized approach to test the role of CAF in developing resistance to drug/combination(s). Here, we designed a new model system to test the function of patient-derived endometrial CAFs in resisting the growth-inhibitory effect of a chemotherapy drug, paclitaxel. Paclitaxel is routinely used along with platinum agents to treat advanced endometrial cancers, and the administration of paclitaxel (weekly) as a single agent to patients with recurrent or metastatic disease is the most commonly practiced approach in clinics [28,29] (https://www.cancer.org/ accessed on 12 December 2022).

We observed a discrepancy in DiO-CAFs’ fluorescence intensities between the 2D and 3D formats after 7 days of HyCC. As expected, the fluorescence intensities of DiO-CAFs in 3D formats were increased with time in contrast to the fluorescence intensities of DiO-CAFs in 3D formats. The primary components of matrigel are four major basement membrane ECM proteins, including laminin and collagen IV. We used BD Matrigel™ Basement Membrane, which contains laminin, collagen IV, TGF-beta, an epidermal growth factor, an insulin-like growth factor, and a fibroblast growth factor. Since 3D culturing involved using the growth media with matrigel, it is theoretically possible that the CAFs growth pattern was additionally supported by matrigel, with special reference to TGF-beta and fibroblast growth factor. We also noted a characteristic morphological formation of CAFs, both TCAFs and NACFs, when the cultures were in 3D matrigel. Our novel model will provide a unique option to test the resistance to a drug or a combination in a primary CAF derived from the post-surgical tissues of either particular patient planning to undergo prospective adjuvant therapy or post-surgery surveillance.

The power of our model is that, though it was developed and tested using endometrial CAFs, it can be adapted to any organ type of cancer(s) for testing the development of resistance to adjuvant therapy drugs/combination(s) post-surgery. Recently, we studied the role of patient-derived primary ovarian CAFs in resisting the anti-angiogenic effect of lenvatinib on HUVEC cells [9]. Our ex vivo two-cell HyCC-based model of drug resistance provides a cost-effective and laboratory-friendly way to establish CAF, and then test the functions of fibroblasts in each patient with advanced and recurrent endometrial cancers and other solid tumors. Our enzymatic digestion-independent method of establishment of CAF needs a simple method of culturing, followed by CAF characterization via standard flow cytometry, ICC, and qRT-PCR. The HyCC model is a vital fluorescent dye-based 3D matrigel assay that can be performed in any standard laboratory, including a community-based cancer center such as the Avera Cancer Institute. Thus, the model can serve as a patient-specific real-time testing modality for the function of fibroblasts in patients with advanced and recurrent endometrial cancers. The single most significant limitation of the model is that the number of CAFs derived from the available patient tissue limits the experimental setup. Hence, the testing power of the model is tissue-limited and can only be applied to test the development of resistance to adjuvant therapy. We have tried to use 2–3 biopsy tissue cores to test the amount of the volume of the starting tissue sample, with no success. Additionally, in our study, some tumor tissue samples did not yield CAFs. It is possible that the status of CAF is a determinist factor here, as most of these tissues were obtained from patients with grade 1/stage I diseases. Additionally, as the starting material is a post-surgical resected tumor tissue sample, the model will not be applicable to neoplasms of inaccessible organ types, such as in some central nervous system tumors, unless a tumor can be obtained following surgical resection.

As the results of current CAF-directed clinical trials will be incorporated into the current literature, we will begin to become more confident about a CAF-inclusive treatment plan to manage the advanced form of the disease, especially in the context of the development of treatment resistance in solid tumors. Our model system can provide a unique opportunity for the personalized system to test the development of resistance to the same drugs/combinations received by patients as an adjuvant treatment in clinics. During the entire course of the development and management of a tumor, from the preneoplastic stage to the terminal stage of metastasis and resistance, CAFs play an undeniably complex and multifaceted role. Acknowledging the challenges of CAF biology, the contribution of CAF cannot be ignored as a mechanistic determinant of resistance and clinical outcome of a treatment modality, chemotherapy, radiotherapy, targeted therapy, and immunotherapy. The study of CAF biology is on the cusp of the next wave of discovery that will facilitate the tailored targeting of the cancer types in combination with targeting tumor cells to optimize the clinical benefit and disease management.

## 5. Patent

The study presented in the MS is part of a patent application (United States Patent and Trademark Office); application number 16/875,910.

## Data Availability

Not applicable.
